# The value of arterial pressure waveform cardiac output measurements in the radial and femoral artery in major cardiac surgery patients

**DOI:** 10.1186/s12871-017-0334-2

**Published:** 2017-03-14

**Authors:** A. van Drumpt, J. van Bommel, S. Hoeks, F. Grüne, T. Wolvetang, J. Bekkers, M. ter Horst

**Affiliations:** 1 Department of Anesthesiology, Erasmus Medical Center, PO Box 2040, 3000 CA Rotterdam, The Netherlands; 2 Department of Intensive Care Adults, Erasmus Medical Center, Rotterdam, The Netherlands; 3 Department of Cardiothoracic Surgery, Erasmus Medical Center, Rotterdam, The Netherlands

**Keywords:** Cardiac output, Transpulmonary thermodilution, Pulse contour analysis, Fluid responsiveness, Agreement, Trending ability

## Abstract

**Background:**

A relatively new uncalibrated arterial pressure waveform cardiac output (CO) measurement technique is the Pulsioflex-ProAQT® system. Aim of this study was to validate this system in cardiac surgery patients with a specific focus on the evaluation of a difference in the radial versus the femoral arterial access, the value of the auto-calibration modus and the ability to show fluid-induced changes.

**Methods:**

In twenty-five patients scheduled for ascending aorta, aortic arch replacement, or both we measured CO simultaneously by transpulmonary thermodilution (COtd) and by using the ProAQT® system connected to the radial (COpR), as well as the femoral artery catheter (COpF). Hemodynamic data were assessed at predefined time points; from incision until 16 h after ICU admission.

**Results:**

In total 175 (radial) and 179 (femoral) pairs of CO measurement were collected. The accuracy of COpR/COpF was evaluated showing a mean bias of −0.31 L/min (±2.9 L/min) and -0.57 L/min (± 2.8 L/min) with percentage errors of 49 and 46% respectively. Trending ability of the ProAQT® device was evaluated; the four quadrant concordance rates in the radial and femoral artery were 74 and 75% and improved to 77 and 85% after auto-calibration. The mean angular biases in the radial and femoral artery were 6.4° and 6.0° and improved to 5° and 3.3° after auto-calibration. The polar concordance rates in the radial and femoral artery were 65 and 70% and improved to 76 and 84% after auto-calibration. Considering the fluid-induced changes in stroke volume(SV), the coefficient of correlation between the changes in SVtd and SVp was 0.57 (*p* < 0.01) in the radial artery and 0.60 (*p* < 0.01) in the femoral artery.

**Conclusions:**

The ProAQT® system can be of additional value if the clinician wants to determine fluid responsiveness in cardiac surgery patients. However, the ProAQT® system provided inaccurate CO measurements compared to transpulmonary thermodilution. The trending ability was poor for COpR but moderate for COpF. Auto-calibration of the system did not improve accuracy of CO measurements nor did it improve the prediction of fluid responsiveness. However, the trending ability was improved by auto-calibration, possibly by correcting a drift over a longer time period.

## Background

Goal-directed therapy (GDT) is a practice to achieve optimal tissue perfusion by optimizing hemodynamic parameters with responses to fluid resuscitation and administration of inotropic and vasopressor medication. GDT uses flow-directed hemodynamic parameters such as stroke volume (SV) and the related cardiac output (CO). GDT has been shown to improve patient outcome by reducing morbidity and the length of stay on the intensive care unit in high-risk patients undergoing major surgery [1, 2].

In the peri-operative setting, clinicians are looking for a system able to detect changes in CO over a longer period of time to access the effect of fluid loading and inotropic medication (trending ability). Traditionally, repeated thermodilution (td) is used for CO assessment in an intrapulmonary or transpulmonary fashion (i.e. PiCCO system). The latter system is validated in cardiac surgery patients [3–6].

Several uncalibrated waveform analysis CO monitors have been developed. Without thermodilution these devices do not require central venous access and a special arterial thermistor catheter in a large artery, and as such are less invasive and more easily applicable. The uncalibrated CO monitors have been investigated under various clinical conditions. These validation studies showed conflicting results regarding their reliability [7–11].

A relatively new uncalibrated CO monitoring device is the ProAQT® sensor connected to the Pulsioflex monitor (Pulsion, Maquet Getinge Group, Germany), hereafter called the ProAQT® system. With this uncalibrated system, it is relatively easy to promote the standard arterial catheter pressure system to CO trend monitoring.

Studies comparing the ProAQT® system with calibrated systems are rare in cardiac surgery patients [12–14]. A limitation in these studies is the fact that the ProAQT® sensor was connected to the femoral artery catheter while the ProAQT® is supposed to be easily used at the radial site.

The ProAQT® manufacturer advises to use the auto-calibration mode that works by manual input. This is based on software that estimates the aortic compliance and systemic vascular resistance. Only one study investigated this auto-calibration mode [13].

The aim of this study is to validate the ProAQT® CO monitoring in major cardiac surgery patients prone to hemodynamic fluctuations in the postoperative period. For this purpose, the accuracy and the ability to track changes in CO over time (trending ability) and in response to a standard fluid challenge (fluid responsiveness) in radial and femoral artery are compared with the PiCCO system.

We hypothesize that the uncalibrated pulse contour CO measurement has a good trending ability, independent of the arterial catheter site (i.e. radial or femoral artery). In addition, the effect of the internal auto-calibration modus will be assessed.

## Methods

### Patients and anesthesia

After obtaining approval from the Ethics Committee of our hospital (MEC 2014-210) and written informed consent from all patients, 25 patients were enrolled from August 2014 to February 2016. This prospective, non-interventional study was performed at the Departments of Anesthesiology and Intensive Care Medicine of the Erasmus Medical Center (Rotterdam, the Netherlands). Patients were included if they were scheduled for distal ascending aorta or aortic arch replacement and deep hypothermic circulatory arrest was expected to be necessary during surgery. Prolonged cardiopulmonary bypass time and deep hypothermic circulatory arrest causes more hemodynamic fluctuations in the postoperative period at the intensive care unit. We hypothesized the clinician would have most benefit of extra cardiac output monitoring in this specific patient group. Dual arterial pressure monitoring in this type of cardiac surgery is standard care. Exclusion criteria were age < 18 years old, emergency surgery, intracardiac shunts and a contra-indication for femoral artery catheterization.

Anesthesia, cardiac surgery and postoperative intensive care management were performed according to institutional standards. Before anesthesia was induced, an arterial catheter (Flowswitch 20G, BD, USA or Leadercath 20G, Vygon, UK) was inserted into the right radial artery. If this artery was not accessible, a catheter was placed in the right brachial artery. Thereafter anesthesia was induced and a 16-cm 9,5 F five-lumen central venous catheter (Arrow International Inc, Teleflex Medical, Reading, USA) was inserted into the right internal jugular vene. A PiCCO cold injectate delivery system (Pulsion, Maquet Getinge Group, Germany) used for intermittent transpulmonary thermodilution was connected to the proximal lumen and a pressure transducer was connected to measure central venous pressure. A 20-cm 5 F thermistor-tipped arterial catheter (Pulsiocath, Maquet Getinge Group, Germany) was introduced via the left femoral artery. This catheter was connected to a pressure transducer, which was connected to the PiCCO monitor as well as to another transducer system that was connected to a Pulsioflex monitor (Pulsion, Maquet Getinge Group, Germany). The arterial pressure catheter in the radial artery was connected to a second Pulsioflex monitor.

Hemodynamic interventions, such as fluid loading and cardiovascular drug support, were given at the discretion of the attending anesthesiologist, in particular based on intra-operative transesophageal echocardiography and by the intensivist in the postoperative period.

### ProAQT® system

The ProAQT® system estimates CO with arterial pressure waveform analysis. It needs the input of patient data (age, gender, weight, height). To calculate CO, the ProAQT® software uses the algorithm derived from the PiCCO Pulse-Contour-Analysis technology. The algorithm is based on the relationship between area under the arterial pressure curve and stroke volume and a value quantifying vessel compliance and peripheral resistance. Vessel compliance is estimated based on demographic patient data and arterial resistance is calculated from arterial waveform characteristics [15, 16]. The system collects data with a high sampling frequency of 250 Hz to provide beat-to-beat CO monitoring.

### The reference method

For adequate CO determination and the adjustment of individual aortic compliance we used the PiCCO calibration method by transpulmonary thermodilution [17].

### Study protocol

At each time point, three CO measurements were recorded simultaneously: COtd (using thermodilution technique with the PiCCO system), COpR (using the ProAQT® system connected to the radial artery) and COpF (using the ProAQT® system connected to the femoral artery). The thermodilution measurements (COtd) were performed in triplicate with a sodium chloride 0.9% solution (20 mL, 4–6 °C) via the central venous catheter [18]. The average of three measurements was used for analysis. If the global end-diastolic volume readings varied by more than 10%, one more thermodilution measurement was performed and the extreme one was excluded. During thermodilution measurements, COpR and COpF were registered by a dedicated observer, before and after activation of the internal auto-calibration modus.

Measurements were performed at pre-defined time points: after induction of anesthesia just before incision (T0), after extracorporeal circulation and stabilized hemodynamics (T1), after sternal closure (T2), on arrival to ICU (T3), before a fluid challenge within 6 h after admission on the ICU (T4), immediately after this fluid challenge (T5), 8 h after arrival on the ICU (T6) and 16 h after arrival on the ICU (T7). The fluid challenge was defined as a bolus of more than 250 mL of fluid administered in less than 15 min. All study measurements were performed under stable hemodynamic conditions; therefore no hemodynamic interventions were performed during the CO collection period.

### Statistical analysis

For data collection and statistical analysis, we used SPSS (version 21; SPSS Inc., Chicago, IL, USA) and an Excel spreadsheet (Microsoft Office Excel 2007, Microsoft Corp, USA). To generate the plots, we used the open source statistical program RStudio using packages (http://cran.r-project.org/) and Prism 6.0 (Graphpad Software, La Jolla, California, USA). Based on the data distribution visually inspected and tested with the Shapiro-Wilk test, results were expressed as mean (standard deviation) or median (interquartile range). Repeated hemodynamic data were analysed by a test of contrasts with a paired t test or the Wilcoxon signed rank test, depending on data distribution, with a Bonferroni correction for repeated testing.

To evaluate the accuracy of the ProAQT® device we described the agreement between the paired data of COp and COtd using the Bland-Altman analysis with correction for multiple measurements per subject [19]. Subsequently, the percentage error (PE) was calculated as 1.96xSD of the bias of the tested method divided by the mean CO of the tested and the reference method × 100% [20]. An acceptable PE and acceptable agreement is recently discussed [21–23]. It depends on the error of the reference method and the precision that is clinically acceptable. The true error of the thermodilution reference method is unknown. We agreed with an acceptable PE of maximal 30% set by Critchley et al. based on an estimated precision of 20% for the thermodilution reference method and based on a clinically acceptable precision of less than 1 L/min for a mean cardiac output of around 5 L/min [24].

To investigate the ability of the ProAQT® device to detect serial changes in CO (i.e. trending ability), we used the four quadrant plot and the polar plot approach [25, 26]. In the four-quadrant plots, the ProAQT® ∆CO is plotted against the reference ∆CO. The four quadrant concordance rate is defined as the percentage of the number of data points that fall into 1 of the 2 quadrants of agreement. At the center of the plot, data tend to be randomly and not depending on the degree of agreement between the reference and test method. Therefore, exclusion zones for central data points are advised. In our study we chose an exclusion zone of 0.75 L/min, which is 12,5% of the mean CO [25]. A limitation in all CO comparison studies is the lack of well-defined cutoff values for good, acceptable and poor trending ability based on concordance rates [27, 28]. As a guide, we agreed with the best available cut off value which stated a concordance rate with the use of an exclusion zone of more than 92% indicates good trending ability [25, 26].

The polar plot analysis describes the vector of CO change as an angle to the line of identity (x = y). In case this angle is zero or 180°, the agreement of the two CO readings is 100%. The magnitude of change in CO is represented as the average of the reference (PiCCO) and the test (ProAQT®) cardiac output. We used the recommended smaller exclusion zone of 10% in the polar plots [25]. In our study this was set on 0.5 L/min. Two statistical variables were derived from the polar data: (1) the mean polar angle or angular bias, which is the average angle between all polar data points and the horizontal polar axis. The angular bias provides an assessment of how well the test method is calibrated compared with the reference method. Good calibration can be considered to exist when the angular bias is no greater than 5% [25]. (2) The polar concordance rate, which is based on the proportion of data points that lie within 30° of the polar axis. A polar concordance rate of more than 95% indicates good trending [25]. Because exclusion zones are still a matter of debate and might hinder good comparison between different studies, we computed both the concordance rates with and without excluding central zone data.

Because Stroke Volume (SV) is a more pure predictor of fluid responsiveness instead of CO we visualized changes in SV before and after a fluid challenge. We deliberately did not use the above described statistical methods to describe fluid induced changes because of the small sample size (*n* = 22). Comparison of fluid-induced changes in SV between thermodilution and the ProAQT® measurement was made by the Spearman coefficient (chosen based on distribution of SV data). Correlation coefficients were compared by using the Fisher transformation. The change in SV in the ProAQT® system was calculated by using the auto-calibrated SV data before fluid bolus and both the auto- and non-calibrated SV data after the fluid bolus. We defined fluid responsiveness as an increase in SV of more than 10% [29].

## Results

Twenty-six patients were assesed for eligibility. During the total inclusion period 4 patients met the inclusion criteria but were not assessed for eligibility because of absence of appropriate personel to perform the measurements. No patient declined to participate, one patient was excluded due to vascular prothesis in both femoral arteries. Characteristics of the 25 enrolled patients and surgical procedures are shown in Table 1. Of 196 COtd measurements collected, 13 were excluded because of aortic insufficiency prior to surgical correction (aortic valve replacement). No other CO data were excluded. There were 6 missing COtd measurements due to human error. Two patients had an arterial catheter placed in the brachial artery because the radial artery was not accessible. In 3 patients the radial artery catheter failed during the ICU period resulting in a few missing measurements. A total of 175 (radial) and 179 (femoral) pairs of CO measurement were collected. The median collection time for each data pair including tripled COtd measurement was 149 (129–177) seconds.

**Table 1:** Demographic data and surgical procedure characteristics

Patient	
Age (y)	67(9)
Male/Female	14/11
Weight(kg)	85(17)
Body Mass index (kg/cm2)	28(4)
Diabetic	3
Length of ICU stay (days)	1(1–2.3)
No valve dysfunction pre-operative	12
LVF	
Good	20
Mildly decreased	5
Poor	0
Surgical procedure	
Bentall procedure	2
Ascending aorta replacement	2
Hemi- or aortic arch replacement	12
Bentall and hemi-aortic arch replacement	9
Degree of hypothermia	
18–24 °C	20
24–30 °C	1
30–37 °C	4
Operation time (min)	458(90)
Cardiopulmonary bypass time (min)	226(64)

Data are presented as the number, mean (SD) of median (25th–75th percentile)

Hemodynamic data are summarized in Table 2. The CO values of the reference method (COtd) ranged from 2.6 to 9.9 L/min with an average of 5.9 (1.5) L/min for the total study period. All patients received low dose inotropic support with dobutamine and/or bolus enoximone and low dose vasopressor support with noradrenaline the first hours after CPB. Vasopressor support with noradrenaline was titrated based on a target blood pressure.

**Table 2:** Hemodynamic data

	T0 Before incision	T1 After CPB	T2 After sternal closure	T3 Arrival in ICU	T4 Before fluid bolus	T5 After fluid bolus	T6 8 h in ICU	T7 16 h in ICU
HR (pbm)	59 (14)	78 (13) *	80 (15) *	78 (12) *	80 (14) *	80 (12) *	77 (11) *	71 (11) *
MAP (mmHG)	71 (69–81)	73 (65–85)	69 (66–82)	80 (74–86)	75 (72–78)	78 (73–82)	82 (76–84)	78 (68–89)
CVP (mmHG)	12 (5–14)	17 (5–21) *	15 (5–20) *	12 (5–14)	8 (5–11)	9 (5–13)	7 (6–12)	6 (5–9)
SVR PiCCO (dyn/s/cm^−5^)	1460 (1208–1837)	753 (600–894) *	807 (666–955) *	977 (784–1127) *	960 (792–1101) *	899 (803–1127) *	1050 (860–1292) *	1268 (957–1413) *
GEDV PiCCO (ml)	1531 (1300–1981)	1504 (1237–1759)	1450 (1243–1680)	1580 (1165–2031)	1307 (1159–1697)	1349 (1131–1864)	1268 (1146–1874)	1194 (1113–1857)
EVLW PiCCO (ml)	592 (475–733)	489 (406–618) *	525 (461–635) *	611 (486–776)	544 (368–558)	508 (375–571)	442 (374–564) *	415 (359–726)
SVV PiCCO (%)	11 (8–15)	11 (7–14)	12 (8–15)	14 (8–18)	12 (7–20)	12 (6–15)	13 (8–23)	12 (10–19) *
PPV PiCCO (%)	12 (8–14)	10 (6–13)	10 (7–13)	10 (7–14)	10 (5–15)	10 (4–13)	11 (9–21)	17 (12–19) *
COtd (L/min)	4.3 (1.3)	6.8 (1.4) *	6.2 (1.3) *	5.9 (1.2) *	5.5 (1.4) *	5.9 (1.4) *	5.8 (1.4) *	5.9 (1.7) *
COpR (L/min)	4.2 (1.2)	6.5 (1.6) *	6.2 (1.6) *	6.5 (1.7) *	5.9 (1.5) *	6.3 (1.7) *	6.3 (1.4) *	6.3 (2.1) *
COpF (L/min)	4.3 (1.2)	6.8 (1.7) *	6.4 (1.7) *	6.6 (1.5) *	6.4 (1.7) *	6.6 (2.0) *	6.5 (1.7) *	6.8 (2.1) *

Data are presented as mean (SD) of median (25^th^–75^th^ percentile). *P < (0.05/7) compared with T0 (Bonferroni corrected)

*Abbreviations*: *HR* heart rate, *MAP* mean arterial pressure, *CVP* central venous pressure, *SVR* systemic vascular resistance, *GEDV* global end-diastolic volume, *SVV* stroke volume variation, *PPV* pulse pressure variation, *COtd* cardiac output assessed by transpulmonary thermodilution with the PiCCO system, *COpR* cardiac output assessed by the ProAQT system connected to the radial artery after auto-calibration, *COpF* cardiac output assessed by the ProAQT system connected to the femoral artery after auto-calibration

### Accuracy

**Fig. 1:**
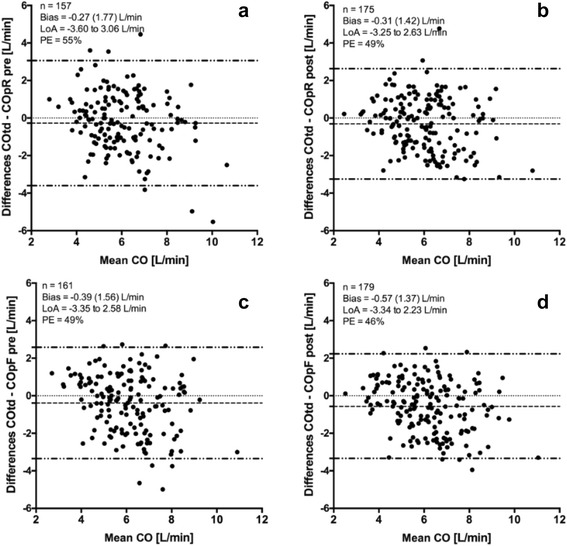
Bland Altman plot. Bland-Altman plots show mean bias and 95% limits of agreement (LoA) in dashed lines. COtd, transpulmonary thermodilution cardiac output assessed by the PiCCO system; COp, pulse contour cardiac output assessed by the ProAQT system. **a** Radial artery data before autocalibration. **b** Radial artery data after autocalibration. **c** Femoral artery data before autocalibration. **d** Femoral artery data after autocalibration

Bland-Altman analysis corrected for repeated measurements comparing ProAQT® CO measurements with transpulmonary thermodilution CO measurement revealed a small negative mean bias at almost all measurement points. Internal calibration of the ProAQT® monitor did not significantly change the mean bias nor did it change the LoA. For the radial artery data, the mean bias between COtd and COpR was −0.31 L/min with LoA of ± 2.9 L/min and a PE of 49%. For the femoral artery data the mean bias between COtd and COpF was −0.56 L/min with LoA of ± 2.8 L/min and a PE of 46%, as illustrated in Fig. 1. Bland-Altman analysis is applied to compare the CO measurements of the radial- and the femoral arterial access, both by using the ProAQT® monitor. The mean bias was 0.25 L/min, LoA ± 1.9 L/min and PE 33%.

### Trending ability

**Fig. 2:**
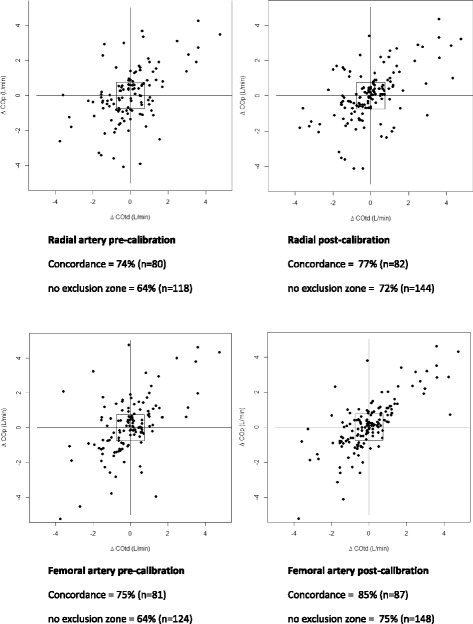
Four Quadrant plot. Four quadrant plots: The serial changes in cardiac output measured with the ProAQT® (∆COp) are plotted against the changes in cardiac output measured by thermodilution (∆COtd). The four quadrant concordance rate, defined as the percentage of the number of data points that fall into 1 of the 2 quadrants of agreement are shown with and without making use of an exclusion zone (0.75 L/min)

**Fig. 3:**
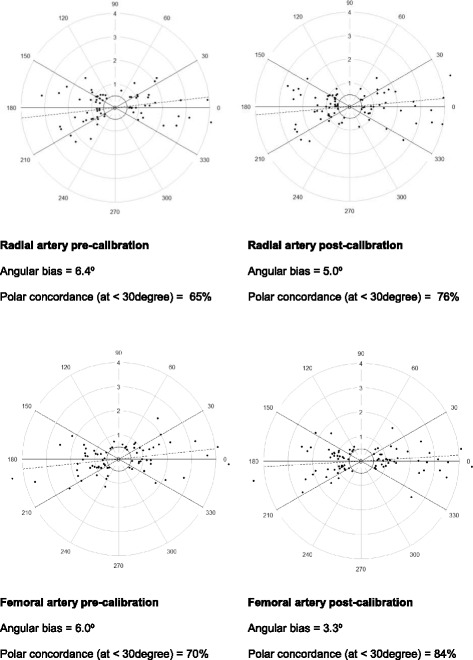
Polar Plot. Polar plots: Polar plot analysis describes the vector of CO change as an angle to the line of identity (x = y). In case this angle is zero or 180°, the agreement of the two CO readings is 100%. The magnitude of change in CO is represented as the average of the reference (PiCCO) and the test (ProAQT®) cardiac output (distance from the center). The exclusion zone is 0.5 L/min. Two statistical variables are shown from the polar data: (1) the angular bias, which is the average angle between all polar data points and the horizontal polar axis. (2) The polar concordance rate, which is the proportion of data points that lie within 30° of the polar axis (*thick lines*)

The four quadrant- and polar plots are shown in Figs. 2 and 3.

The four quadrant concordance rates in the radial and femoral artery were 74 and 75% and improved to 77 and 85% after auto-calibration. In the polar plots, the mean angular bias in the radial and femoral artery were 6.4° and 6.0° and improved to 5° and 3.3° after auto-calibration, respectively. Data points which fell within the 30° limits (i.e. polar concordance rate) in the radial and femoral artery were 65 and 70% and improved to 76 and 84% after auto-calibration.

### Fluid responsiveness

**Fig. 4:**
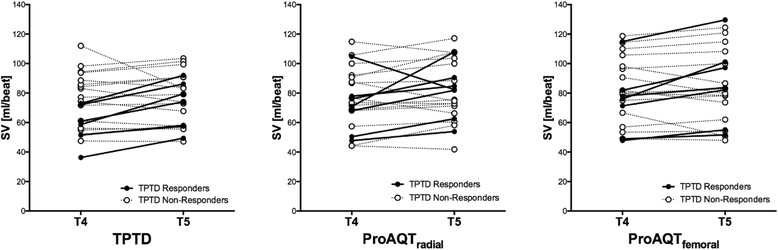
Fluid Responsiveness. Changes in stroke volume (SV) before (T4) and after (T5) a fluid bolus. We defined fluid responsiveness as an increase in SV of more than 10%. The 7 thick lines are correlating with the data of the patients that were fluid responsive measured by transpulmonary thermodilution (TPTD Responders). The ProAQT® fluid responsiveness agreed in most patients based on the pulse contour SV calculation in the radial artery (*middle figure*) and in the femoral artery (*right figure*)

In 22 of 25 patients a fluid challenge was given. Figure 4 shows changes in SV after this fluid bolus. Fluid responsiveness defined as an increase in SV of more than 10% was found in 7 out of 22 patients based on the increase in SV computed by transpulmonary thermodilution (SVtd). The ProAQT® fluid responsiveness (SVp) agreed in 20 out of 22 patients based on the SV calculation in the radial artery and agreed in 21 out of 22 patients based on the SV calculation in the femoral artery.

The coefficient of correlation between the fluid induced changes in SVtd and SVp was 0.57 (*p* < 0.01) in the radial artery data and 0.60 (*p* < 0.01) in the femoral artery data. Auto-calibration of the ProAQT® system after fluid administration deteriorated correlation in SV changes in radial artery data and remained comparable in femoral artery data (0.28 (*p* = 0.20) and 0.48 (*p* = 0.02), respectively).

## Discussion

Based on the results of our study, we conclude that the uncalibrated pulse contour CO measurement is unreliable to estimate an absolute value of CO compared to the transpulmonary thermodilution technique in major cardiac surgery patients. In time, the overall trending ability was moderate to poor. The auto-calibration modus did not improve accuracy but it significantly improved trending ability of the device. The best performance in trending ability was seen when the ProAQT® monitor was connected to the femoral artery catheter with the use of the auto-calibration modus. However, the assessment of fluid responsiveness showed overall good agreement.

### Accuracy

Our results showed broad LoA and a high PE in the radial as well as the femoral artery CO measurement. Smetkin et al. used a similar study protocol but their CO measurements with the ProAQT® showed a clinical acceptable accuracy with much smaller LoA [12]. They observed a slight underestimation of the ProAQT® CO measurements. Our data, except from the measurement just after CPB, showed an overestimation of the ProAQT® with a mean bias of 0.3 L/min in the radial artery and of 0.6 L/min in the femoral artery. Possible explanation for these conflicting results could be a difference in systemic vascular resistance after CPB and hypothermia. Most of our patients underwent aortic arch replacement having a long CPB time and deep hypothermic arrest, while the patients in the study of Smetkin underwent off pump coronary artery surgery and remained normothermic.

Our study as well as two other validation studies found a PE far above the 30% limits of acceptable agreement which were recommended by Critchley et al. [24]. Monnet et al. measured before and after administration of fluids or norepinephrine in intensive care patients and observed an unacceptable high PE of 59% [13]. Broch et al. evaluated the accuracy of the ProAQT® before and after CPB. They also observed an unacceptable high PE of 62% before and 49% after CPB [14]. There are some statistical issues in the interpretation of PE. Manach et al. stated the choice of up to 30% PE to be acceptable limits of agreement is only realistic if the averaged thermodilution reference method has a good precision in comparison with the real and variable CO, which cannot be measured. Furthermore, PE calculation is influenced by small sample size [23]. However, also if we do not focus on the calculated PE but on the absolute differences between ProAQT® CO data and our reference CO data, we suspect that these absolute differences could lead to a relevant different treatment strategy in clinical use. Therefore, we conclude that the ProAQT® cannot reliably estimate absolute CO values in comparison to thermodilution.

### Trending ability

An advantage of the ProAQT® device in daily ICU practice is that it does not require insertion of a special arterial catheter or transducer system, it is easy to install and results are obtained within a few minutes. For clinical use, it is especially relevant if the system has the ability to track changes in CO over a longer period of time and thus has the ability to track changes in CO after administration of a fluid bolus (i.e. fluid responsiveness). Despite the lack of clearly defined cut-off values for definition of good, acceptable and poor trending ability, we found the overall concordance rates in the four quadrant as well as in the polar plot to be moderate to low. We conclude a poor trending ability of the ProAQT® compared to transpulmanary thermodilution in the radial artery and moderate trending ability in the femoral artery. Our conclusion is in agreement with the study of Smetkin. The authors conclude the ability of the ProAQT® to follow trends in CO is poor [12]. The study of Broch et al. revealed conflicting results. Their four quadrant plots showed concordance rates indicating acceptable trending ability, but their polar plot analysis showed a poor trending ability [14]. However, the polar plot method has some important drawbacks, as described by Saugel [27]. Besides the fact that the plot is not easy to interpret, the use of exclusion zones could lead to the exclusion of relevant data points that are most discordant. Despite the above-mentioned discussion we agreed at this moment, the four quadrant plot in combination with the polar plot method to be the best available method to evaluate trending ability.

### Fluid responsiveness

The trending ability of the ProAQT® after fluid administration is remarkably better than the overall trending ability of our CO data. The ProAQT® reliably followed the changes in SV induced by a fluid bolus. These findings are in agreement with the study of Monnet, in which it was also concluded that the ability of the ProAQT® to track fluid-induced changes was reliable [13]. This is an important finding for clinical use. It is relatively easy to enhance the standard arterial catheter system with ProAQT® monitoring. With the ability to reliably track changes in SV after administration of a fluid bolus, these extra hemodynamic data facilitate decision making between inotropes, vasopressors and fluid therapy. Especially if other post-operative measurements, for example lactate, central venous saturation, urine production, blood pressure or echocardiography measurements, are inconclusive.

### Femoral versus radial measurement site

According to the manufacturer, the ProAQT® algorithm enables the use of arterial signals from different detection sites. Besides demographic patient data, the monitor asks for input of the vascular access site. Because the pulse pressure is a result of the SV and the properties of the vascular system between heart and the measurement site, the software must somehow correct for differences in vascular compliance and vascular resistance to make a reliable CO calculation. The present study showed that the accuracy of CO measurements via the radial and femoral artery using the ProAQT® sensor was not significantly different but the trending ability was better when connecting the ProAQT® sensor to the femoral artery catheter. The CO value from the femoral artery catheter was on average 0.25 L/min higher. These results are in agreement with other studies evaluating another type of pulse contour analysis system using both a radial and a femoral arterial catheter [10, 30, 31]. The difference between radial and femoral data might be caused by variability in a central-to-radial arterial pressure gradient as recently has been analyzed by Fuda et al. [32]. In this study radial, femoral and aortic pressures were measured in 73 cardiac surgery patients. Normally, radial pulse pressure is wider than aortic pulse pressure. This central-to-radial pulse amplification depends on gradual stiffening of the central elastic artery toward the peripheral muscular arteries. Interestingly, almost half of cardiac surgery patients using CPB developed a reversed central-to-radial pressure gradient. Furthermore, the gradient appearance was dynamic in time and prolonged aortic clamping time was an independent predictor for the development of this reversed gradient. Our patients had a long CBP and aortic clamping time and this might explain the better performance of the pulse contour radial artery measurements in the off-pump patient group [12].

A possible confounder in our study is the fact that the ProAQT® software cannot correct for changes in pulsewave characteristics caused by placement of central aorta vascular prosthesis. Another limitation is the fact that we used a more centrally placed 20 cm long PiCCO arterial catheter in the femoral artery. When we evaluated the ProAQT® connected to the radial and femoral artery data we did not only compare the ProAQT® sensor and software corrections but also the different catheter types. However, if we take this consideration into account, our data suggest that the trending ability of the pulse contour ProAQT® measurements connected to the less invasive radial artery cannulation is not as good as the pulse contour ProAQT® measurements when connecting the sensor to the thermodilution femoral catheter.

### Auto-calibration

Auto-calibration is a software mode that estimates the aortic compliance and systemic vascular resistance based on a combination of patient data and specific characteristics of the shape of the pressure waveform.

Based on these data the software can make a recalculation of the pulse contour analysis data. This calibration is not performed automatically, but works on demand by manual input. We questioned if auto-calibration of the ProAQT® system improved accuracy and trending ability. Our data showed that auto-calibration improved trending ability in both the radial and the femoral artery but it did not improve the accuracy of the CO measurements. Conversely, Monnet et al. found that auto-calibration did not improve the trending ability [13]. A reason for these conflicting findings might be the fact that Monnet et al. studied changes in CO in a shorter period of time (10 min versus several hours in our study). In our study, 20 out of 25 patients received hypothermic arrest (18 –24 °C). In the postoperative period the measured systemic vascular resistance increased over a longer period of time. These conflicting results could be an argument for further studies determining the effect of the auto-calibration modus over longer periods of time and related to changes in vascular resistance.

## Conclusions

The ProAQT® system can be of added value if the clinician is questioning fluid responsiveness in major cardiac surgery patients. However, we observed inaccurate absolute CO measurements with this pulse contour monitoring system compared to transpulmonary thermodilution. Trending ability of the ProAQT® was poor when the device was connected to the radial artery and improved to moderate trending ability when it was connected to the femoral artery. Auto-calibration of the system did not improve the accuracy of CO measurements and did not improve prediction of fluid responsiveness but it did improve the trending ability, possibly by correcting a drift over a longer time period.

## Abbreviations

CO: Cardiac output
COpF: Cardiac output measurement with the ProAQT® sensor connected to femoral artery catheter
COpR: Cardiac output measurement with the ProAQT® sensor connected to radial artery catheter
COtd: Cardiac output measurement with transpulmonary thermodilution with the PiCCO system
CPB: Cardiopulmonary bypass
GDT: Goal-directed therapy
ICU: Intensive care unit
LoA: Limits of agreement
PE: Percentage error
SV: Stroke volume
SVtd: Stroke volume measurement with transpulmonary thermodilution with the PiCCO system
td: Thermodilution

## References

[1] Aya HD, Cecconi M, Hamilton M, Rhodes A (2013). Goal-directed therapy in cardiac surgery: a systematic review and meta-analysis. Br J Anaesth.

[2] Cecconi M, Corredor C, Arulkumaran N, Abuella G, Ball J, Grounds RM (2013). Clinical review: Goal-directed therapy-what is the evidence in surgical patients? The effect on different risk groups. Crit Care (London, England).

[3] Hadian M, Pinsky MR (2006). Evidence-based review of the use of the pulmonary artery catheter: impact data and complications. Crit Care (London, England).

[4] Buhre W, Weyland A, Kazmaier S, Hanekop GG, Baryalei MM, Sydow M (1999). Comparison of cardiac output assessed by pulse-contour analysis and thermodilution in patients undergoing minimally invasive direct coronary artery bypass grafting. J Cardiothorac Vasc Anesth.

[5] Ostergaard M, Nielsen J, Rasmussen JP, Berthelsen PG (2006). Cardiac output--pulse contour analysis vs. pulmonary artery thermodilution. Acta Anaesthesiol Scand.

[6] Hadian M, Kim HK, Severyn DA, Pinsky MR (2010). Cross-comparison of cardiac output trending accuracy of LiDCO, PiCCO, FloTrac and pulmonary artery catheters. Crit Care (London, England).

[7] Slagt C, Malagon I, Groeneveld AB (2014). Systematic review of uncalibrated arterial pressure waveform analysis to determine cardiac output and stroke volume variation. Br J Anaesth.

[8] Broch O, Renner J, Gruenewald M, Meybohm P, Schottler J, Steinfath M (2012). A comparison of third-generation semi-invasive arterial waveform analysis with thermodilution in patients undergoing coronary surgery. TheScientificWorldJOURNAL.

[9] Desebbe O, Henaine R, Keller G, Koffel C, Garcia H, Rosamel P (2013). Ability of the third-generation FloTrac/Vigileo software to track changes in cardiac output in cardiac surgery patients: a polar plot approach. J Cardiothorac Vasc Anesth.

[10] Hofer CK, Button D, Weibel L, Genoni M, Zollinger A (2010). Uncalibrated radial and femoral arterial pressure waveform analysis for continuous cardiac output measurement: an evaluation in cardiac surgery patients. J Cardiothorac Vasc Anesth.

[11] Suehiro K, Tanaka K, Funao T, Matsuura T, Mori T, Nishikawa K (2013). Systemic vascular resistance has an impact on the reliability of the Vigileo-FloTrac system in measuring cardiac output and tracking cardiac output changes. Br J Anaesth.

[12] Smetkin AA, Hussain A, Kuzkov VV, Bjertnaes LJ, Kirov MY (2014). Validation of cardiac output monitoring based on uncalibrated pulse contour analysis vs transpulmonary thermodilution during off-pump coronary artery bypass grafting. Br J Anaesth.

[13] Monnet X, Vaquer S, Anguel N, Jozwiak M, Cipriani F, Richard C (2015). Comparison of pulse contour analysis by Pulsioflex and Vigileo to measure and track changes of cardiac output in critically ill patients. Br J Anaesth.

[14] Broch O, Carbonell J, Ferrando C, Metzner M, Carstens A, Albrecht M (2015). Accuracy of an autocalibrated pulse contour analysis in cardiac surgery patients: a bi-center clinical trial. BMC Anesthesiol.

[15] Chew MS, Aneman A (2013). Haemodynamic monitoring using arterial waveform analysis. Curr Opin Crit Care.

[16] Montenij LJ, de Waal EE, Buhre WF (2011). Arterial waveform analysis in anesthesia and critical care. Curr Opin Anaesthesiol.

[17] Reuter DA, Huang C, Edrich T, Shernan SK, Eltzschig HK (2010). Cardiac output monitoring using indicator-dilution techniques: basics, limits, and perspectives. Anesth Analg.

[18] Monnet X, Persichini R, Ktari M, Jozwiak M, Richard C, Teboul JL (2011). Precision of the transpulmonary thermodilution measurements. Crit Care (London, England).

[19] Bland JM, Altman DG (2007). Agreement between methods of measurement with multiple observations per individual. J Biopharm Stat.

[20] Critchley LA, Critchley JA (1999). A meta-analysis of studies using bias and precision statistics to compare cardiac output measurement techniques. J Clin Monit Comput.

[21] Peyton PJ, Chong SW (2010). Minimally invasive measurement of cardiac output during surgery and critical care: a meta-analysis of accuracy and precision. Anesthesiology.

[22] Hapfelmeier A, Cecconi M, Saugel B (2016). Cardiac output method comparison studies: the relation of the precision of agreement and the precision of method. J Clin Monit Comput.

[23] Le Manach Y, Collins GS (2016). Disagreement between cardiac output measurement devices: which device is the gold standard?. Br J Anaesth.

[24] Critchley LA (2011). Bias and precision statistics: should we still adhere to the 30% benchmark for cardiac output monitor validation studies?. Anesthesiology.

[25] Critchley LA, Yang XX, Lee A (2011). Assessment of trending ability of cardiac output monitors by polar plot methodology. J Cardiothorac Vasc Anesth.

[26] Critchley LA, Lee A, Ho AM (2010). A critical review of the ability of continuous cardiac output monitors to measure trends in cardiac output. Anesth Analg.

[27] Saugel B, Grothe O, Wagner JY (2015). Tracking changes in cardiac output: statistical considerations on the 4-quadrant plot and the polar plot methodology. Anesth Analg.

[28] Saugel B, Wagner JY (2016). Innovative noninvasive hemodynamic monitoring: curb your enthusiasm after initial validation studies and evaluate the technologies clinical applicability. J Clin Monit Comput.

[29] Marik PE, Monnet X, Teboul JL (2011). Hemodynamic parameters to guide fluid therapy. Ann Intensive Care.

[30] Vasdev S, Chauhan S, Choudhury M, Hote MP, Malik M, Kiran U (2012). Arterial pressure waveform derived cardiac output FloTrac/Vigileo system (third generation software): comparison of two monitoring sites with the thermodilution cardiac output. J Clin Monit Comput.

[31] Schramm S, Albrecht E, Frascarolo P, Chassot PG, Spahn DR (2010). Validity of an arterial pressure waveform analysis device: does the puncture site play a role in the agreement with intermittent pulmonary artery catheter thermodilution measurements?. J Cardiothorac Vasc Anesth.

[32] Fuda G, Denault A, Deschamps A, Bouchard D, Fortier A, Lambert J (2016). Risk factors involved in central-to-radial arterial pressure gradient during cardiac surgery. Anesth Analg.

